# Animal Viruses: Molecular Biology

**DOI:** 10.3201/eid1405.080077

**Published:** 2008-05

**Authors:** Brian W.J. Mahy

**Affiliations:** * Centers for Disease Control and Prevention, Atlanta, Georgia, USA

**Keywords:** animal viruses, molecular biology, book review

In this multi-author work, Mettenleiter and Sobrino have compiled 10 chapters that describe what is currently known about the molecular biology of some of the most interesting viruses of veterinary importance, from the tiny circovirus of pigs (1,800 nt of single-stranded DNA) to the highly complex African swine fever virus (≈200,000 nt pairs of double-stranded DNA). It is fitting that the first chapter describes foot-and-mouth disease virus, which was the first animal virus to be described by Loeffler and Frosch, who worked in Griefswald-Insel Riems, where Mettenleiter is currently the president of the Friedrich-Loeffler Institut. All 10 chapters are written by experts in their respective fields. Mettenleiter is a coauthor for a chapter about herpesviruses, whereas Sobrino is a coauthor for one on foot-and-mouth disease virus. Polly Roy wrote a chapter about bluetongue virus, one of the major threats to the livestock industry worldwide, which recently emerged in Europe, perhaps because global warming has allowed the *Culicoides *vector to survive and overwinter. Another chapter is about Hendra and Nipah viruses, which are newly emerging in Southeast Asia and Australia. There are also informative chapters on arteriviruses, coronaviruses, and pestiviruses. Finally, in 1 chapter, Hans-Dieter Klenk and colleagues write about viruses of birds, including avian influenza. They discuss the molecular mechanism of pathogenesis and host range for the virus everyone fears may give rise to the next influenza pandemic.

The book would have been improved by including a chapter on paramyxoviruses, of which rinderpest virus of cattle and Newcastle disease virus of birds are 2 important examples. But, overall, this compilation is excellent and is rounded off by a scholarly and provocative epilogue about animal virology by Esteban Domingo and Marian C. Horzinek. It is almost worth buying the book for these 10 pages alone.

